# Corals and Their Potential Applications to Integrative Medicine

**DOI:** 10.1155/2014/184959

**Published:** 2014-03-13

**Authors:** Edwin L. Cooper, Kyle Hirabayashi, Kevin B. Strychar, Paul W. Sammarco

**Affiliations:** ^1^Laboratory of Comparative Neuroimmunology, Department of Neurobiology, David Geffen School of Medicine at UCLA, Los Angeles, CA 90095-1763, USA; ^2^Annis Water Resources Institute, Grand Valley State University, Muskegon, MI 49441-1678, USA; ^3^Louisiana Universities Marine Consortium (LUMCON), 8124 Highway 56, Chauvin, LA 70344-2110, USA

## Abstract

Over the last few years, we have pursued the use and exploitation of invertebrate immune systems, most notably their humoral products, to determine what effects their complex molecules might exert on humans, specifically their potential for therapeutic applications. This endeavor, called “bioprospecting,” is an emerging necessity for biomedical research. In order to treat the currently “untreatable,” or to discover more efficient treatment modalities, all options and potential sources must be exhausted so that we can provide the best care to patients, that is, proceed from forest and ocean ecosystems through the laboratory to the bedside. Here, we review current research findings that have yielded therapeutic benefits, particularly as derived from soft and hard corals. Several applications have already been demonstrated, including anti-inflammatory properties, anticancer properties, bone repair, and neurological benefits.

## 1. What Are Corals?

Corals have served as an excellent target taxon for bioprospecting [1–4]. The earth's surface is covered by ~70% water and contains 80% of all life found on the planet [5]. It is no wonder then that the ocean has been and still is a source of food, let alone a vast source of therapeutic molecules. In our case, corals will be the subject of our investigation. Corals (Phylum: Cnidaria, Class:* Anthozoa*) can generally be categorized into hard, soft, or gorgonian-type organisms. Hard corals, called Scleractinian corals, are generally hermatypic, the types that build reefs, with which most people are familiar. Soft corals or octocorals are generally flexible and do not produce the rigid structure characteristic of hard corals [6]. Gorgonian corals are also flexible, but their skeletal systems consist of a horny substance called gorgonin. Some of these coral types contain symbiotic microalgae, genus* Symbiodinium*, and are generally called zooxanthellae [7]. These coral types tend to reside in shallow waters of tropical and subtropical locales.

**Figure 1:**
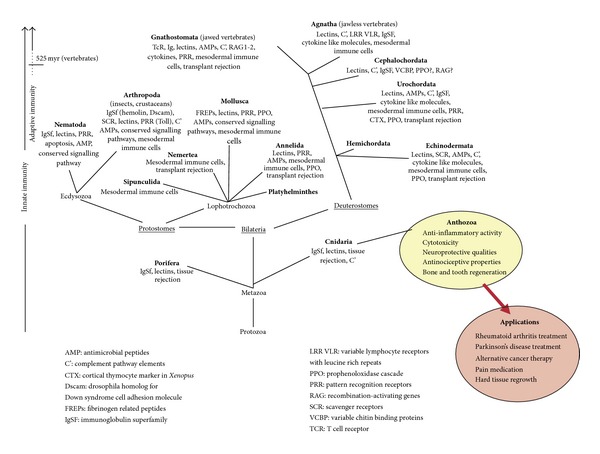
An evolutionary map indicating where corals (*Anthozoa*) lie and some of the therapeutic benefits they exert. Adapted from [11].

Through the extensive work of Metchnikoff, we now know of cellular and humoral immunity as well as the importance of invertebrate organisms in immunologic research [8, 9]. The revelation of a division between innate and adaptive immunity came much later. The innate immune system is natural, nonspecific, nonanticipatory, nonclonal, germ line, and for which there is no evidence that such a system possesses the capacity to build memory from past exposures. Conversely, the adaptive immune system is acquired, specific, anticipatory, clonal, and somatic and possesses the capacity to build memory from past exposures [10]. Adaptive or acquired immunity has always been associated with vertebrates while the innate immune system is generally thought to have originated in the invertebrates [11]. We propose immune-related products as sources of therapeutic molecules (Figure 1).

The discipline of coral immunology has not been vastly studied and is not completely understood. While many mechanisms associated with other organisms have been identified and examined, the general system of coral immunology still requires further investigation [12]. One of the mechanisms that has been demonstrated extensively is alloimmune memory, or reaction to and rejection of cells and molecules from members of the same species. For example,* Montipora verrucosa*, a hard (Scleractinian) coral, actually displays a limited form of memory during analyses of transplantation responses [13, 14]. Alloimmune memory in this coral seems to be due to varying histocompatibility markers that serve as identification badges of self/nonself. We will now turn to the therapeutic benefits of certain coral species.

## 2. Corals and Inflammation

Perhaps disorders most related to the immune system are those of inflammation. Inflammation is a physiological protective mechanism that is activated by the immune system upon encountering foreign invaders in an attempt to remove the adverse stimuli and begin healing the organism. Diterpenes are one specific class of molecules isolated from corals that have been shown to exhibit therapeutic benefits, specifically anti-inflammatory. Three diterpenes isolated from* Eunicea fusca*, a gorgonian coral native to Florida, were found to have superior anti-inflammatory effects to those of indomethacin, a commonly used anti-inflammatory medication [15]. Another diterpene isolated from the same species of coral, fucoside E, showed the same anti-inflammatory activity as well as antimicrobial activity [16]. Sinularin, a molecule that we will discuss later in greater detail, blocks pathways that increase the severity of neuroinflammation (see Table 1 for a more extensive list of therapeutic molecules isolated from corals) [17].

**Figure 2:**
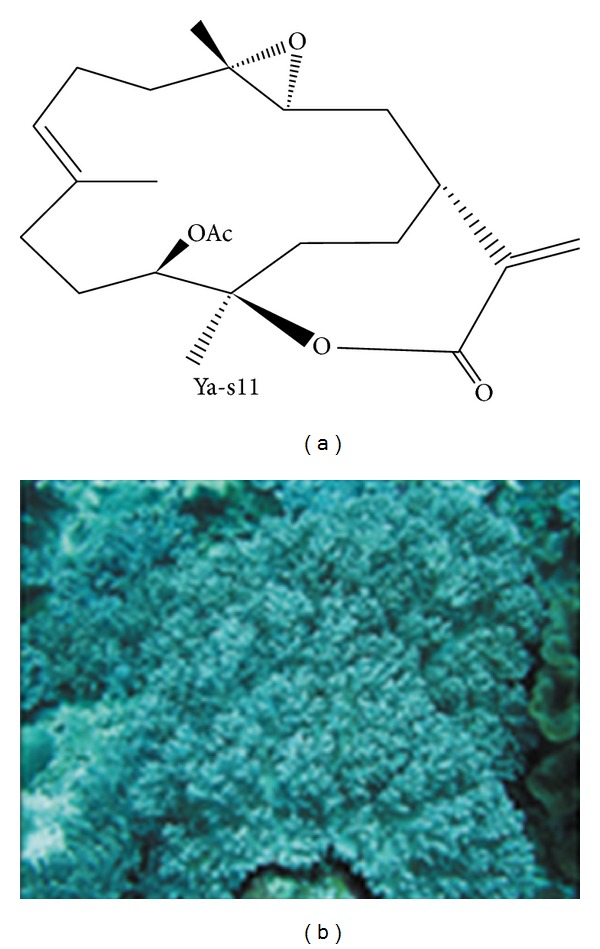
The chemical structure of 11-epi-sinulariolide acetate (Ya-s11) and the coral from which it is isolated,* Sinularia querciformis*. From [18].

**Figure 3:**
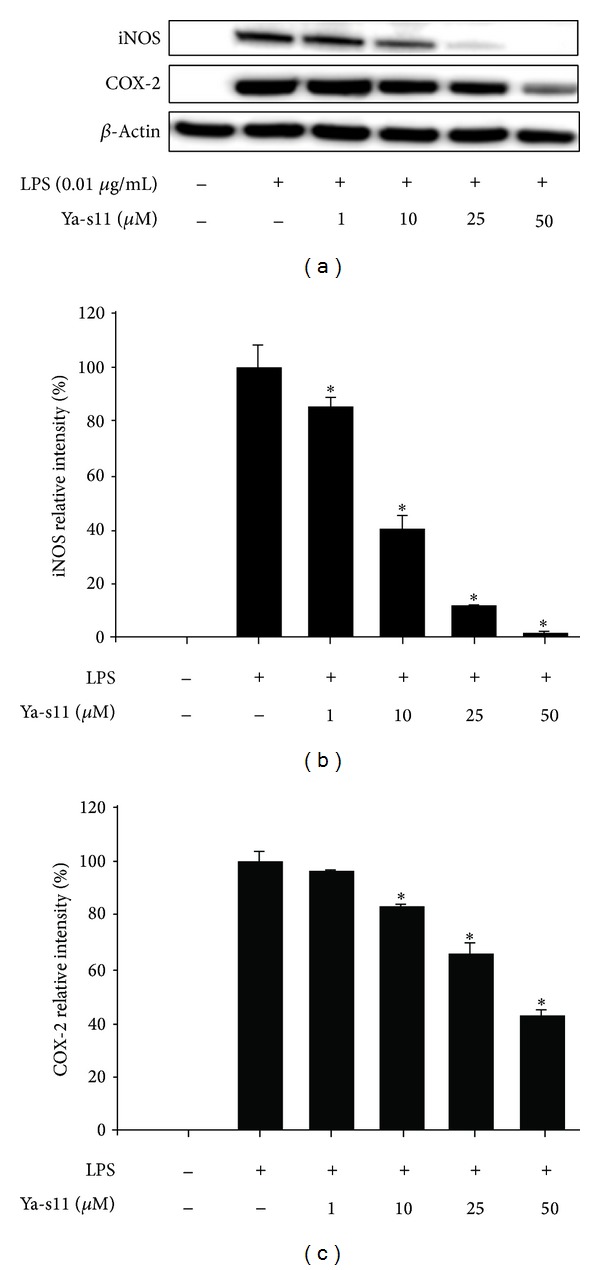
*In vitro* effects of Ya-s11 on production of iNOS and COX-2, proinflammatory proteins. From [18].

**Figure 4:**
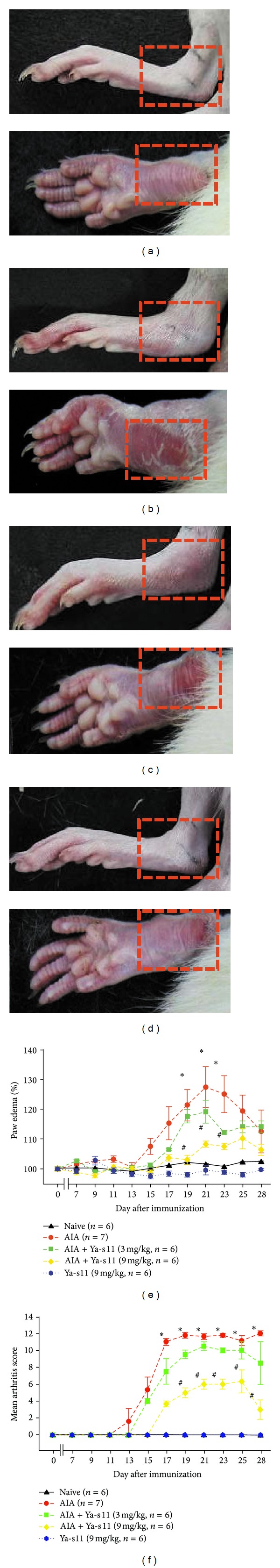
Pictorial and graphical demonstrations of the attenuation of AIA symptoms in rats. From [18].

**Figure 5:**
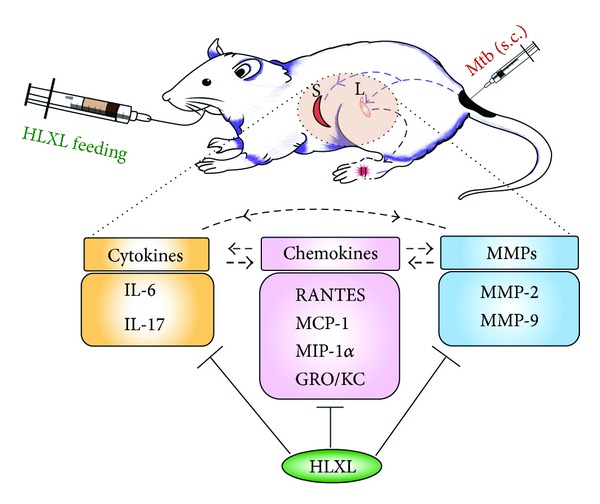
A schematic of how herbal supplements were administered and evaluated as a treatment for AIA in rats. From [19].

One particular clinical application that has proven to be especially promising in relation to coral natural products is arthritis. Rheumatoid arthritis is a chronic inflammatory disease that causes joint destruction. In a recent article published in* PLoS*, 11-epi-sinulariolide acetate (Ya-s11), a known cembrane-type compound, was isolated from the soft coral,* Sinularia querciformis*, and evaluated in its anti-inflammatory potency* in vitro* as well as in adjuvant-induced arthritis (AIA) in rats (Figure 2) [18]. AIA is the typical murine equivalent to human rheumatoid arthritis used in experiments to test treatment possibilities. Ya-s11 was shown to strongly inhibit the production of proinflammatory proteins iNOS and COX-2 in murine macrophages (Figure 3). In the AIA mice, Ya-s11 severely reduced the effects of arthritis by inhibiting migration of inflammatory cells to joints and preventing bone destruction (Figure 4). The authors concluded that this molecule may be a source of treatment for humans with rheumatoid arthritis. To draw a correlation here, there have also been other demonstrations of AIA attenuation in rats through the use of herbal products (Figure 5) [19]. Although the investigators used different methods for evaluating the effectiveness, the point we are making is that there are various potential natural treatments, whether plant or animal, being tested for the treatment of arthritis. Next, we will examine anticancer effects that some coral natural products exert.

## 3. Corals and Cancer

### 3.1. The Role of* Sinularia* in Cancer

One particular genus of corals possesses a natural metabolite which has demonstrated significant anticancer ability. They are species of the soft coral genus* Sinularia*. Goto et al. isolated an agglutinin called sinularian from this species and found that it agglutinated experimental targets, for example, rabbit erythrocytes and murine leukemia cells, but not sheep or human erythrocytes [20]. Incidentally responses to foreign erythrocytes are a common approach used in analyzing the innate immune system of invertebrates. Moreover, Wright et al. isolated various compounds from corals of this genus, including a new nitrogenous diterpene, new and known lobanes, and known cembranes [21]. The lobanes and cembranes were tested for anticancer activity against three human cancer cell lines and showed a 50% inhibition of tumor growth. Not only does this coral's product exhibit anticancer activity, but it also exerts pronounced anti-inflammatory responses.

**Figure 6:**
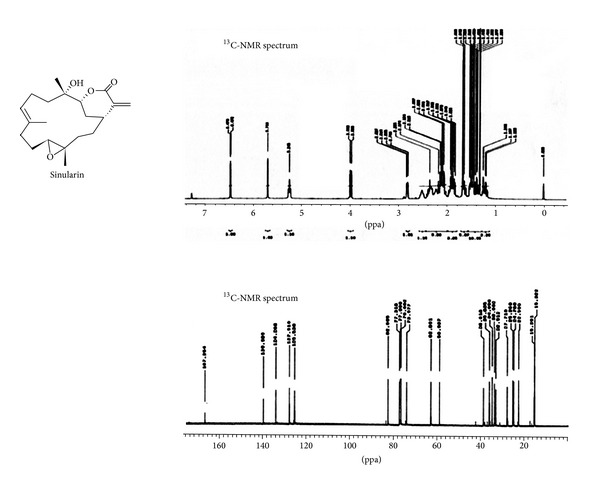
The chemical structure and ^13^C-NMR spectra of sinularin, a potential anticancer molecule isolated from* Sinularia*. From [20].

**Figure 7:**
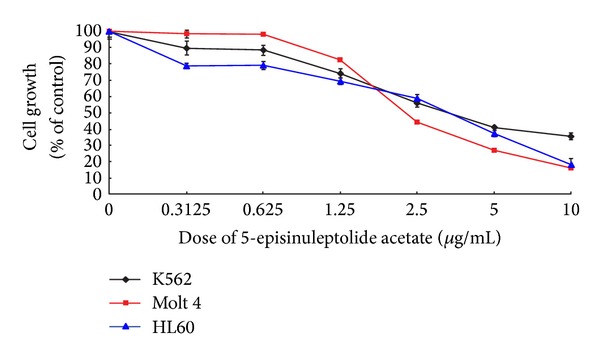
5EPA activity against K562, Molt 4, and HL60 cell lines. From [24].

The innate immune system's property of phagocytosis gave rise to the well-understood common inflammatory system and its responses. Novel steroids isolated from* Sinularia crassa* were found to downregulate expression of proinflammatory proteins and showed cytotoxic activity against human liver cancer cells [22]. Another molecule isolated from* Sinularia *sp., sinularin, demonstrated anticancer activity* via* proapoptotic factors (Figure 6) [23]. Apoptosis refers to one type of programmed cell death leading to particular cell conformational changes and death. 5-episinuleptolide acetate (5EPA), a norcembranoidal diterpene, isolated from* Sinularia *sp., has demonstrated cytotoxicity against many cell lines including K562, Molt 4, and HL 60, with HL 60 being the most sensitive to this treatment* via* Hsp90 inhibition (Figure 7) [24]. Hsp90 inhibition leads to apoptosis or cell death in the leukemia cells.

### 3.2. Other Species of Soft Corals with Anticancer Properties

Other species, besides those in the genus* Sinularia*, have also demonstrated anticancer potential. One in particular, 13-acetoxysarcocrassolide, isolated from the soft coral* Sarcophyton crassocaule *exerted cytotoxic activity against bladder cancer cells [25]. Wang and Duh isolated six novel cembranolides called michaolides and a known cembranolide called lobomichaolide from the soft coral* Lobophytum michaelae* and determined that they possessed not only antitumor capabilities, but also activity against cytomegalovirus (HCMV), related to herpesviruses [26].

Another genus of corals that has proven particularly generous with its demonstrations of marine natural products is* Nephthea* [27]. Wang, Puu, and Duh isolated three new steroids, nebrosteroids Q, R, and S, from the soft coral* Nephthea chabrolii* and tested its anticancer and antiviral activity. They found that these three steroids did exhibit cytotoxicity against the P-388 cell line (mouse lymphocytic leukemia) but did not exhibit antiviral activity against HCMV.

## 4. A Focus on Hard Tissue Therapies

### 4.1. Bone Repair

In addition to the anti-inflammatory and anticancer properties outlined above, corals and their products have been shown to exert curative potential for metabolic deficits as well. The naturally occurring calcium within the aragonite found in scleractinian hard corals, and the calcite found within the soft octocorals, when administered in conjunction with zeolite, a microporous mineral, assists in protecting against and reversing bone loss in mice that have been placed into an artificial, induced menopausal state [28] (mice, like most mammals, have an estrous cycle). This same effect was observed in rabbits when used with human platelet-rich plasma [29]. Green et al. have highlighted the importance of the skeletal matrices of marine invertebrates for bone regeneration [30].

### 4.2. Restoration of Dental Deformities

Related to the potential for bone repair discussed above is bioprospecting being performed within corals as it pertains to dental treatment. Figueiredo et al. mention that their interest in this application has been increasing due to the low supply and difficulty of using human-derived substitutes for certain products used in dental procedures [31], making this a necessary and promising area of research regarding this untapped resource. They assert, however, that the coral skeleton in its unrefined form remains impractical because of its high degeneration (dissolution) rate. They argue that the potential benefit of coral skeleton may reside in refined coral that is modified (strengthened chemically) to increase its longevity and integrity. A particularly interesting experiment was performed which revealed that osteogenic bone marrow stromal cells used with coral scaffolds could be effective in repairing mandibular defects in canines [32].

## 5. The Nervous System: Neuroprotective Compounds

One of the highly prospective treatment modalities that can be added to the benefits of soft corals is the potential for abbreviating neurological deficits. Chen et al. extracted a neuroprotective compound, 11-dehydrosinularolide, from a coral that they believe could be used to treat Parkinson's disease [33]. Parkinson's disease is a highly debilitating neurodegenerative disorder that leads to severe impairment in the central nervous system and the necessity for special care. Most of the treatments that exist for Parkinson's disease only partially improve the symptoms and do not treat the source of the disease itself. Despite treatment using current pharmaceuticals, progression of the disease continues, with the patient losing motor skills and standard functions. Two mechanisms which impact the pathogenesis of Parkinson's disease, however, are inflammation and apoptosis [34]. Proinflammatory iNOS and COX-2 are two proteins that are well known to be associated with inflammation and are common indices and markers when testing for anti-inflammatory activity. Hoang et al. found that neuronal NOS and COX-2 caused DNA damage in a mouse model of Parkinson's disease [35]. The next logical step in trying to find a treatment for this debilitating disease is to find an agent that will prevent the inflammation induced by iNOS and COX-2. In Chen et al.'s experiment, investigators found that administration of 11-dehydrosinulariolide significantly reduced expression of iNOS and COX-2, with iNOS being almost entirely eliminated. It was also demonstrated to halt apoptosis, another factor thought to be associated with Parkinson's disease. This molecule provides highly promising potential for this severely incapacitating disease.

Not only have corals yielded molecules that confer neuroprotection, but also that reduce neuropathic pain. Austrasulfone, which is isolated from the soft coral* Cladiella australis*, demonstrated neuroprotective qualities* via* anti-inflammatory pathways (reduction of iNOS and COX-2) [36]. It also showed potency in reducing neuropathic pain and slowing progression of atherosclerosis and multiple sclerosis models in rats. Another molecule that exerts anti-inflammatory effects by reducing iNOS and COX-2 is capnellene, which is isolated from the soft coral,* Capnella imbricata* [37]. In conjunction with the anti-inflammatory properties, capnellene also exhibited antinociceptive properties. Nociception refers to the neurological pathway by which the body perceives pain caused by tissue-damaging stimuli. Therefore, this compound holds the potential not only to reduce inflammation causing or worsening neurodegenerative disorders, but also to reduce any pain that may be associated as a symptom.

## 6. Hypertensive Treatments

While most of the research done pertaining to corals and therapeutic applications has yielded results in cancer treatment and bone repair, other applications have also been identified. For example, coral sand, which is biogenic, is generated by the erosion of scleractinian corals. It has been used as a silicon source to reduce blood pressure as well as to improve the expression of genes that increase cardiovascular health in hypertensive rats [38].

## 7. Comparing Corals with Therapeutic Properties of Other Invertebrates

The reason that we and others are interested in corals as potential sources of medicinal molecules for humans is the vast amount of progress already made with other invertebrate organisms. Investigators have found an abundance of promising evidence for earthworms specifically as treatment modalities for inflammation, cancer, and coagulatory disorders [39–43]. Earthworms have also been used historically as a source of nutrition [44, 45]. Careful analyses do not seem to reveal that corals, as nutritious sources, afford this same sustenance as food. This is probably due to the small amount of tissue available in relation to their skeletal materials, even in soft corals. Current results clearly indicate certain medicinal qualities. Moreover, cytotoxicity has been demonstrated by molecules isolated from tunicates, another invertebrate [46]. With this large amount of evidence, it is definitely clear we should be looking towards other invertebrates in our bioprospecting endeavors.

## Figures and Tables

**Table 1:** Bioactive compounds with medicinal qualities.

Genus	Species	Molecule	Benefit	Author
*Sinularia *	*flexibilis *	Flexibiliquinone Flexibilin D	Anti-inflammation	Lin et al. 2013 [47] Hu et al. 2013 [48]
	*querciformis *	11-epi-Sinulariolide Acetate Sinularin	Anti-inflammation and anti-bone-loss Antinociception and antineuroinflammation	Lin et al. 2013 [18] Huang et al. 2012 [17]
	*gaweli *	5*α*,8*α*-Epidioxysterol	Cytotoxicity	Yen et al. 2013 [49]
	*crassa *	Crassalone A	Cytotoxicity	Cheng et al. 2012 [50]
	*granosa *	9,11-Secosterol	Cytotoxicity and anti-inflammation	Huang et al. 2012 [51]
	sp.	5-Epsinuleptolide acetate (5EPA)	Cytotoxicity	Huang et al. 2013 [24]
*Scleronephthya *	*gracillimum *	Sclerosteroids	Cytotoxicity and anti-inflammation	Fang et al. 2013 [52]
*Cladiella *	*krempfi *	Kremptielins	Cytotoxicity and anti-inflammation	Tai et al. 2013 [53]
*Paraminabea *	*acronocephala *	Paraminabic acid	Cytotoxicity and anti-inflammation	Chao et al. 2013 [54]
*Capnella *	*imbricata *	Capnellenes	Antineuroinflammation and antinociception	Jean et al. 2009 [37]
*Lemnalia *	*cervicomi tenuis *	Lemnalol	Anti-inflammation and antinociception	Lee et al. 2013 [55]
*Echinomuricea *	sp.	Echinoclerodane A	Cytotoxicity and anti-inflammation	Cheng et al. 2012 [56]
*Nephthea *	*chabrolii *	Nebrosteroids Q, R, and S	Cytotoxicity	Wang et al. 2013 [27]
